# IQdb: an intelligence quotient score-associated gene resource for human intelligence

**DOI:** 10.1093/database/bat063

**Published:** 2013-09-11

**Authors:** Lei Kong, Lu Cheng, Li-ya Fan, Min Zhao, Hong Qu

**Affiliations:** ^1^Center for Bioinformatics, State Key Laboratory of Protein and Plant Gene Research, College of Life Sciences, Peking University, Beijing 100871, P.R. China and ^2^Software Service Delivery Excellence, IBM China Research Lab, Beijing, 100193 China

## Abstract

Intelligence quotient (IQ) is the most widely used phenotype to characterize human cognitive abilities. Recent advances in studies on human intelligence have identified many new susceptibility genes. However, the genetic mechanisms involved in IQ score and the relationship between IQ score and the risk of mental disorders have won little attention. To address the genetic complexity of IQ score, we have developed IQdb (http://IQdb.cbi.pku.edu.cn), a publicly available database for exploring IQ-associated human genes. In total, we collected 158 experimental verified genes from literature as a core dataset in IQdb. In addition, 46 genomic regions related to IQ score have been curated from literature. Based on the core dataset and 46 confirmed linked genomic regions, more than 6932 potential IQ-related genes are expanded using data of protein–protein interactions. A systematic gene ranking approach was applied to all the collected and expanded genes to represent the relative importance of all the 7090 genes in IQdb. Our further systematic pathway analysis reveals that IQ-associated genes are significantly enriched in multiple signal events, especially related to cognitive systems. Of the 158 genes in the core dataset, 81 are involved in various psychotic and mental disorders. This comprehensive gene resource illustrates the importance of IQdb to our understanding on human intelligence, and highlights the utility of IQdb for elucidating the functions of IQ-associated genes and the cross-talk mechanisms among cognition-related pathways in some mental disorders for community.

**Database URL**: http://IQdb.cbi.pku.edu.cn.

## Introduction

Human intelligence refers to a set of cognitive abilities, such as thinking, remembering, reading, learning, problem solving and using language. The high genetic heterogeneity of intelligence poses an enormous challenge for understanding molecular mechanisms for cognition. Intelligence quotient (IQ) is the most widely used phenotype for characterizing human intelligence in psychometric studies. It is not surprising that IQ score is consistently associated with a number of mental disorders such as schizophrenia, autism, depression and anxiety (1–3). Although genetic epidemiology of the relationship between IQ score and the risk of related mental disorders becomes increasingly clear with various lines of studies, there are no substantial achievements to contribute to understanding the molecular mechanisms underlying human intelligence and relevant mental disorders.

As a quantitative trait, the heritability behind an observed IQ score is due to complex genetic interactions between multiple genes of small effect sizes (4–6). Genetic association studies have identified many candidate genes for human intelligence; however, many candidates fail to be replicated between studies and populations (4). Additionally, current genetic predisposition information is scattered in literature and, to date, there has been no systematic collection and analysis. Hence, there is no detailed investigation on the common molecular mechanisms between IQ score and the risk of related mental disorder. Development of a more comprehensive gene resource is really desired to gain a more complete molecular picture for intelligence and relevant disorders.

In this article, we present the IQdb, an IQ-associated gene database for ongoing development of genes relevant to intelligence and serving as a reference dataset for understanding the mechanisms of human intelligence. The resultant gene list, preferably in IQdb with additional functional and genetic information, including gene association study, family-based linkage study, genome-wide association study and other functional studies, would be a valuable resource for the community. In addition, our systematic pathway and disease enrichment analyses reveal that the IQ-associated genes enriched in multiple signal events are involved with many cancers and mental disorders. To the best of our knowledge, IQdb is the first example of an integrated and comprehensive gene resource that helps to elucidate the relationship between IQ score and genetic risk factors in mental disorders. Our collection could have profound implications for the diagnosis, treatment and prevention of some intelligence-related mental disorders.

## Data Annotations

### Collection of core dataset, experimental verified candidate genes

**Figure 1.**
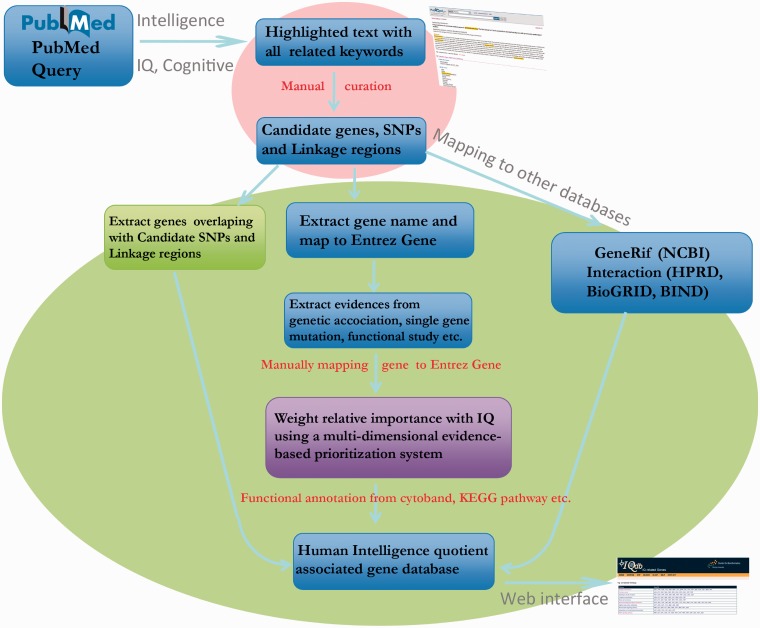
Pipeline for collection, expansion and annotation of IQ-associated genes.

As shown in Figure 1, this comprehensive collection of gene and genomic information for IQdb was accomplished by curating from published articles using the following four steps:

- An extensive literature search, particularly concerning family-based linkage studies, population association studies, genome-wide association studies and other functional analyses, was conducted through PubMed (on 10 January 2013) using the following search terms: [“intelligence quotient” (Title/Abstract) OR “IQ” (Title/Abstract)] AND [“genome-wide association study” (Title/Abstract) OR “genome wide association study” (Title/Abstract)] OR [“gene” (Title/Abstract) OR “genetic” (Title/Abstract)] OR [“association” (Title/Abstract) OR “linkage” (Title/Abstract)]).
- The retrieved 2307 abstracts were highlighted with query keywords and grouped by the function in Entrez system in Related Articles.
- The 2307 abstracts were read manually to curate the experimental verified candidate genes, single-nucleotide polymorphisms (SNPs) and genomic regions relevant to IQ and other related information such as experimental methods and studied population.
- All the names of experimental verified candidate gene and SNPs were manually mapped to 158 Entrez Gene IDs and 139 SNP IDs. For accuracy, we excluded all negative reports. Finally, we defined the 158 genes as a core dataset with high confidence. In addition, 46 genomic regions were also curated from linkage studies (4). To expand the IQ-associated gene list, we overlapped the genes to these curated 46 genomic regions based on RefSeq gene annotation from UCSC genome browser (7).

### Expanding and ranking candidate genes from genomic regions and protein–protein interactions

The molecular basis underlying IQ score is still unclear because of its high genetic heterogeneity. Classical identification of candidate genes in individual studies often focuses on verifying specific genes/variants predisposing to IQ. Therefore, systematic evaluation and summary of relationship between all candidate genes is rare. In this article, we first expanded the IQ-associated genes based on the core dataset using linked genomic regions and protein–protein interactions. Using a multi-dimensional evidence-based candidate gene prioritization approach (8), the relative importance of each expanded gene was estimated based on the supported evidence from literature, genomics regions and functional roles. For instance, 3898 genes locating in the 46 curated genomic regions were expanded. And 3063 genes that interacted with 158 genes in the core dataset were further introduced from the BioGRID (9), HPRD (10) and BIND (11) databases. Finally, 7090 genes, including the genes in the core dataset, were integrated together as a most comprehensive IQ-associated gene list.

To calculate the relativities of all 7090 genes, a benchmark dataset including 19 IQ-associated genes with positive evidence was compiled from a classical review (4) (Supplementary File 1). Then, we followed a gene prioritization approach (12) to generate a candidate weight matrix pool including d^N ^= 4^3^ weight vectors, where N represents the number of evidence, including literature, linkage regions and interactions, and d = N + 1 represents possible different weights, from 1 to 4 in the weight vectors. A combined score for each gene was then calculated by summing up the products of the scores and the corresponding weights from the three evidence (8). All the 7090 candidate genes, including 19 benchmark genes, were sorted by their combined scores. We selected the optimal weight matrix [4, 1, 1] that gave the 95% benchmark genes the highest rank among the top 5% of all candidate genes. Based on the matrix, we evaluated the relevance of the 7090 introduced genes with IQ score, which was useful for users to get potential genes for further screening.

### Biological function annotations

Extensive functional information has been retrieved and integrated for better understanding the function of the IQ-associated genes, such as cross-links to NCBI Entrez gene (13), OMIM (13), UniProt (14), Ensembl (15) and Gene Ontology (16). Comprehensive mRNA expression profiling data are also collected from BioGPS (17), Allen Brain Atlas (18) and RNA-Seq (19–24). Several popular pathway databases are used to get comprehensive pathway-related information, including BioCyc (25), KEGG Pathway (26), PID Curated (27), PANTHER (28), PID Reactome (29, 30), rate-limiting enzyme database (31), pathway localization database (32) and transporter substrate database (33). Other possible association diseases are also integrated from GAD (gene association database) (34), KEGG Disease (35), FunDO (36, 37), NHGRI (38) and OMIM (13). In addition, the original IQ-related literature references in the NCBI PubMed database are linked to each gene in the core dataset. We shall routinely update the core dataset based on newly published literature and other disease-related databases. An automatic annotation pipeline is implemented to integrate functional information from Gene annotation (39), Gene Ontology annotation, HPRD/BIND/BioGRID interaction annotation, KEGG LIGAND/BioCarta signaling event annotation (40, 41) and OMIM annotation using Perl Script and Swiss knife module (10, 42–44). The result shows the automatic pipeline allows IQ-associated gene annotation to be easily updated when new versions of external databases are available.

## Data Analysis

### Pathway enrichment analysis on the core dataset

So far, gene-set pathway enrichment analyses have been probably the most practical and successful data mining approaches to explore underlying molecular causes of complex diseases (45). In this article, the hypergeometric test was used to calculate whether a given set of object pairs had a different frequency of annotation pairs than that would be expected by chance, and it gave the sample sizes involved and the expected frequency of such pairs. Using all known genes in the human genome as background, we have identified the statistically significant enriched pathways and diseases for core dataset genes in IQdb.

Enriched functional pathways for the 158 genes in the core dataset are mainly related to neuronal function such as cocaine addiction, long-term potentiation, dopamine degradation and neurotransmitter release cycle (Table 1). In addition to neuron-related pathways and neurotransmitter biosynthesis and degradation, the genes were also highly enriched in developmental biology. These results highlight that multiple neurotransmitter-related signaling events are related to cognitive process. As the majority of molecules in these signaling pathways play fundamental roles in response to environment signals, regulating neuronal development and synaptic function, integration of these different signals together is the key step to process information. In summary, the level of complexity of signaling systems involved in cognitive systems stems from the functions of components as fundamental cellular roles.

**Table 1.** The statistically significant enriched pathways of IQ-associated genes in the core dataset from different pathway databases

Pathway	Source	Corrected *P*-value*
Neuronal system	Reactome	4.28E-04
Cocaine addiction	KEGG PATHWAY	3.95E-03
Long-term potentiation	KEGG PATHWAY	9.04E-03
Dopamine degradation	BioCyc	1.88E-02
Developmental biology	Reactome	2.51E-02
Noradrenaline and adrenaline degradation	BioCyc	2.51E-02
Adrenaline and noradrenaline biosynthesis	PANTHER	2.76E-02
Arginine and proline metabolism	KEGG PATHWAY	3.79E-02
Serotonin neurotransmitter release cycle	PID Reactome	4.18E-02
Dopamine neurotransmitter release cycle	PID Reactome	4.18E-02
Neurotransmitter release cycle	PID Reactome	4.18E-02

*The corrected *P*-value was calculated by Fisher exact test followed by Benjamini–Hochberg multiple testing correction using the Ingenuity Pathway Tool.

### Enrichment diseases for the 158 IQ-related genes in the core dataset

As a fundamental role of cognition, it is not surprising that the genes are consistently associated with a number of complex diseases. Although it is difficult to measure how much the IQ score may have contributed to certain diseases based on gene content, it might give a clue that helps to generate hypotheses to examine the potential role of IQ score as a risk factor in relevant disease. A quick disease analysis has revealed that the 158 genes in the core dataset are related to a broad spectrum of human diseases such as various cancers and mental disorders (Table 2). In total, 81 genes are related to psychotic and mental disorders. The mental disorders mainly include schizophrenia, autism, depression, bipolar, obsessive-compulsive disorder and Parkinson’s disease. Plenty of previous reports suggest that early-onset and adult-onset schizophrenia are associated with intellectual deficits (46, 47). However, the underlying common molecular mechanism between schizophrenia and IQ scores is still unknown. In IQdb, 37 genes related to schizophrenia are highly enriched in neurotransmitter metabolism pathways, including ‘Adrenaline and noradrenaline biosynthesis’, ‘Dopamine clearance from the synaptic cleft’ and ‘Arginine and proline metabolism’. These pathways suggest that the early-onset and adult-onset schizophrenia might be related to some compound metabolisms such as dopamine metabolism. Most interestingly, several IQ-related genes are associated with several mental disorders. For instance, SLC6A4 is associated with autistic disorder, schizophrenia, obsessive compulsive disorder, bipolar disorder, personality disorders, affective disorder, attention deficit hyperactivity disorder, suicide, Alzheimer’s disease and depression. Thus, the relationships between common IQ-associated genes and diseases are promising for future biological experiments or replication efforts to discover the underlying common pathways. In summary, IQdb is valuable in discovery of potential candidate genes, pathways and potential cross-talks between mental disorder and intelligence using comprehensive annotation and user-friendly interface. As a first effort to systematically collect and extend candidate IQ-associated genes, IQdb is also useful to better clarify the molecular mechanisms related to human intelligence.

**Table 2.** The top 10 enriched diseases of IQ-associated genes in the core dataset with experimental supports

Disease	Source	Corrected *P*-value*
Behavior disease	FunDO	8.71E-09
Psychotic disorder	FunDO	1.42E-08
Autistic disorder	FunDO	2.98E-07
Cognitive function	GAD	9.38E-06
Schizophrenia	GAD	5.66E-05
Obsessive compulsive disorder	GAD	4.26E-04
Noonan syndrome	KEGG DISEASE	6.58E-04
Other congenital disorders	KEGG DISEASE	1.14E-03
Bipolar disorder	FunDO	3.95E-03
Congenital disorders of development	KEGG DISEASE	4.74E-03

*The corrected *P*-value was calculated by Fisher exact test followed by Benjamini–Hochberg multiple testing correction using the Ingenuity Pathway Tool.

## Interface Development of Database

**Figure 2.**
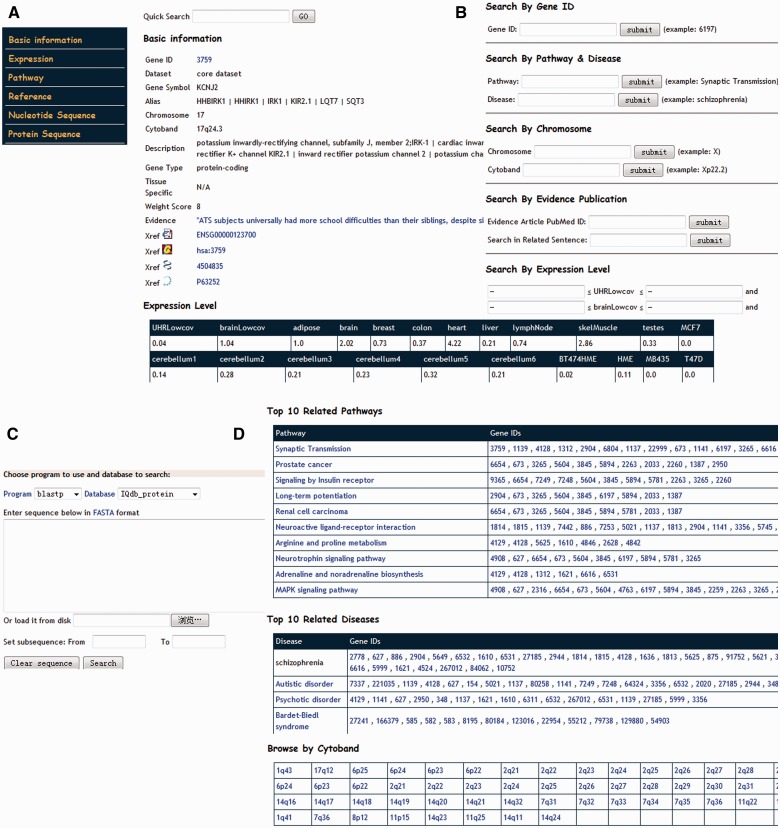
Web interface of IQdb. (**A**) The basic information in each IQ-associated gene page. (**B**) Query interface for text search. (**C**) BLAST search interface for comparing query against all sequences in IQdb. (**D**) Browser interface for genes in top 10 enriched pathways, top 10 enriched diseases and shared cytoband.

All data and information in IQdb are stored in a free, fast and reliable open-source relational database MySQL on a Linux server. Web-based interface to the database is implemented in object-oriented Java, which is a platform-independent language and easy to deploy and update. All the Web applications run under a Tomcat + Apache Web server environment. Based on the JavaServer Pages (JSP) technology, dynamical Web pages for each gene in the database are generated. For genes with different evidence, the comprehensive annotation and links are provided (Figure 2A). Gene expression in various tissues and brain regions is represented in tabular format (Figure 2A). In addition, the original literature to support their association with IQ scores is also complied for the 158 genes in the core dataset. For other expanded genes, literature is compiled from the NCBI GeneRIF database (48), which may be useful for users to judge their potential roles with IQ or other cognitive processes.

IQdb allows users to do text query (Figure 2B), or to run BLAST search against the sequences in IQdb (Figure 2C). To provide a powerful text-based query, six different user-friendly input forms are provided for Entrez Gene ID, pathway and disease annotation, genomic region, literature content and gene expression range in 22 tissues or brains regions. Moreover, a quick full-text search for GeneID, gene symbol or gene alias and publication is on the top right of each page, which is efficient for users to access any data in the database, especially literature-based annotations. In addition, users can browse the data in IQdb in a variety of ways, including significantly enriched pathway, related disease, reported linkage region and chromosome number (Figure 2D). Finally, for any advanced study, IQdb provides all downloadable genetic and population information in a plain text for all the collected 139 SNPs related to IQ.

## Conclusions

IQdb is constructed as a free database and analysis server to enable users to rapidly search and retrieve summarized IQ-associated genes. Enrichment pathway analyses reveal that multiple signal events related to IQ-associated genes are involved in cognitive systems. Central questions should focus on integration of various signaling pathways to process information. In addition, comprehensive disease enrichment analyses interlink IQ-associated genes with many relevant cancers and mental disorders. IQdb is freely available at http://iqdb.cbi.pku.edu.cn.

## Supplementary Data

Supplementary data are available at *Database* Online.

## Funding

This work was supported by the National High-tech 863 Program of China [grant numbers 2006AA02A312, 2008BAI64B01], the National Natural Science Foundation of China [grant number 31171270] and the National Science and Technology Infrastructure Program [grant number 2009FY120100].

*Conflict of interest.* None declared.

## Supplementary Material

Supplementary Data
