# Supplementary material for: IQdb: an intelligence quotient score-associated gene resource for human intelligence
Source: Database (Oxford). 2013 Sep 11;2013:bat063. doi: 10.1093/database/bat063 (PMC3770929; doi:10.1093/database/bat063)
Supplement: Supplementary Data [file supp_2013_bat063_index.html]

Supplementary Data 

# IQdb: an intelligence quotient score-associated gene resource for human intelligence

## Supplementary Data

files

**Files in this Data Supplement:**

- Supplementary Data - xls file
